# Trauma- and Violence-Informed Care Practices in the Emergency Department for Survivors of Intimate Partner Violence

**DOI:** 10.1001/jamanetworkopen.2026.0034

**Published:** 2026-03-03

**Authors:** Gunjan Tiyyagura, Dhatri Abeyaratne, Andrea Asnes, Paula Schaeffer, Marcie Gawel, Nishah Jaferi, Brianna Oakley, Destanee Crawley, Paola Serrechia, Ashley Frechette, Dorene F. Balmer

**Affiliations:** 1 Department of Pediatrics, Yale School of Medicine, New Haven, Connecticut; 2 Department of Emergency Medicine, Yale School of Medicine, New Haven, Connecticut; 3 Hope Family Justice Center, New Haven, Connecticut; 4 Connecticut Coalition against Domestic Violence, Weathersfield; 5 Department of Pediatrics, Children’s Hospital of Philadelphia, Philadelphia, Pennsylvania

## Abstract

**Importance:**

Intimate partner violence (IPV) impacts the health of IPV survivors and their children. Survivors frequently seek care in emergency departments (EDs) and are best served by trauma- and violence-informed care (TVIC), which emphasizes safety, trust building, and collaboration. However, the extent to which ED care aligns with TVIC is underexplored.

**Objective:**

To examine the alignment between TVIC principles and ED care provided to IPV survivors.

**Design, Setting, and Participants:**

This qualitative study was performed in 1 general academic, 1 general community, and 1 pediatric ED using a focused ethnographic approach from November 16, 2022, to June 30, 2024. Participants included a purposive sampling of English- or Spanish-speaking IPV survivors who sought care in the ED. Data included observations of social worker (SW)–survivor interactions and follow-up interviews with IPV survivors within 1 week.

**Exposure:**

ED visit.

**Main Outcomes and Measures:**

Data generated from observation notes and interview transcripts were analyzed deductively (informed by principles of TVIC) and inductively. Codes were created and revised until reaching a stable list, then interpretation of the data was checked with a family violence community advisory board.

**Results:**

Of 31 patient encounters (29 female [94%]; mean [SD] age, 29.0 [8.7] years), 29 included observations and 13 included follow-up interviews. Twenty-one patients presented to the general EDs following IPV-related injuries; most of the 10 encounters in the pediatric ED were disclosed during a child’s medical visit. Six patients preferred Spanish. Findings were clustered into 4 TVIC domains: recognizing the impacts of trauma, prioritizing safety, connectiveness and choice, and fostering development. In recognizing the impacts of trauma, survivors described complex trauma histories that influenced how they navigated IPV and sought help. Safety was prioritized in encounters that were private, calm, and culturally and linguistically responsive, while noise and lack of privacy undermined care. Survivors engaged with SWs who emphasized autonomy and collaboration over directive communication to foster connectedness and choice, particularly when discussing sensitive topics such as child protective services referrals. SWs fostered capacity development by providing resources and handoffs to IPV advocates to address survivors’ basic needs and help them navigate care systems.

**Conclusions and Relevance:**

In this qualitative study of TVIC for IPV survivors in the ED, alignment with TVIC was facilitated by acknowledging past experiences, prioritizing collaboration, and addressing comprehensive needs. Lack of privacy, language barriers, and directive communication styles hindered TVIC alignment. Without TVIC, EDs may retraumatize IPV survivors, undermining their potential to serve as critical points of intervention.

## Key Points

**Question:**

How does care for survivors of intimate partner violence (IPV) and their children align with principles of trauma- and violence-informed care (TVIC) in emergency departments?

**Findings:**

In this qualitative study of 29 observations between social workers and patients experiencing IPV and follow-up interviews, alignment with TVIC was facilitated by acknowledging survivors’ past experiences, prioritizing collaboration, and addressing comprehensive needs. Lack of privacy, language barriers, and directive communication styles hindered TVIC alignment.

**Meaning:**

These findings suggest that enhancing privacy, communication, and collaborative approaches in emergency departments may strengthen trauma- and violence-informed responses to IPV.

## Teaser

This qualitative study examines whether emergency department care practices for survivors of intimate partner violence and their children align with trauma- and violence-informed care principles.

## Introduction

Intimate partner violence (IPV), comprising physical, sexual, or psychological violence or stalking, affects approximately 1 in 3 people in the US.^1^ It leads to short- and long-term impacts on the mental and physical health of survivors and their children.^2,3^ Emergency departments (EDs) are critical access points, with IPV rates between 9% and 37% during a 12-month period, depending on how IPV is ascertained, the sample, and the types of IPV assessed.^4,5,6^ EDs serve socially disadvantaged populations, including uninsured individuals and those with mental illness or substance use disorder, which often accompany IPV.^4,7^ ED visits offer a potent opportunity to support survivors. However, negative interactions can lead survivors to avoid care, decline resources, and lose trust in health care.^8,9,10^

Building on the concept of trauma-informed practice, trauma- and violence-informed care (TVIC) prioritizes safety, collaboration, and capacity building, recognizing that trauma stems from broader systemic inequities such as racism and xenophobia. TVIC emphasizes that survivors’ intersecting marginalized identities, including race, gender, and socioeconomic and immigration status, shape their vulnerability and experiences seeking care.^11,12,13^ Without TVIC, EDs can feel unsafe or even retraumatize IPV survivors, undermining their potential to offer support.

While many EDs endorse TVIC in principle,^11^ the degree to which these principles are implemented is not well understood. A review of 41 studies^8^ found poor adherence to trauma-informed approaches during IPV-related ED encounters. Notably missing from the literature was the experience of survivors with intersecting marginalized identities and those disclosing IPV in pediatric settings. Finally, most studies used interviews, focus groups, or questionnaires to understand survivors’ experiences, but were unable to capture clinical care interactions.

This study examined how well ED care practices for IPV survivors align with TVIC principles. We focused on social worker (SW) consultations, as, unlike the primarily medical evaluations of physicians and nurses, SW encounters involve in-depth discussions of sensitive topics and linkage to community resources, which allowed us to ethically and meaningfully observe behaviors relevant to TVIC. We sought to answer 3 questions: (1) How do ED SWs operationalize TVIC principles in their interactions with IPV survivors? (2) What factors within the ED environment facilitate or hinder TVIC? and (3) How do survivors experience and interpret these interactions?

## Methods

The Institutional Review Board of Yale University approved the study. All participants provided verbal informed consent for observation and written informed consent to follow-up. The Consolidated Criteria for Reporting Qualitative Research (COREQ) checklist guided transparent reporting of researcher reflexivity, study design, and findings. An overview of the study process is provided in the Figure.

**Figure.**
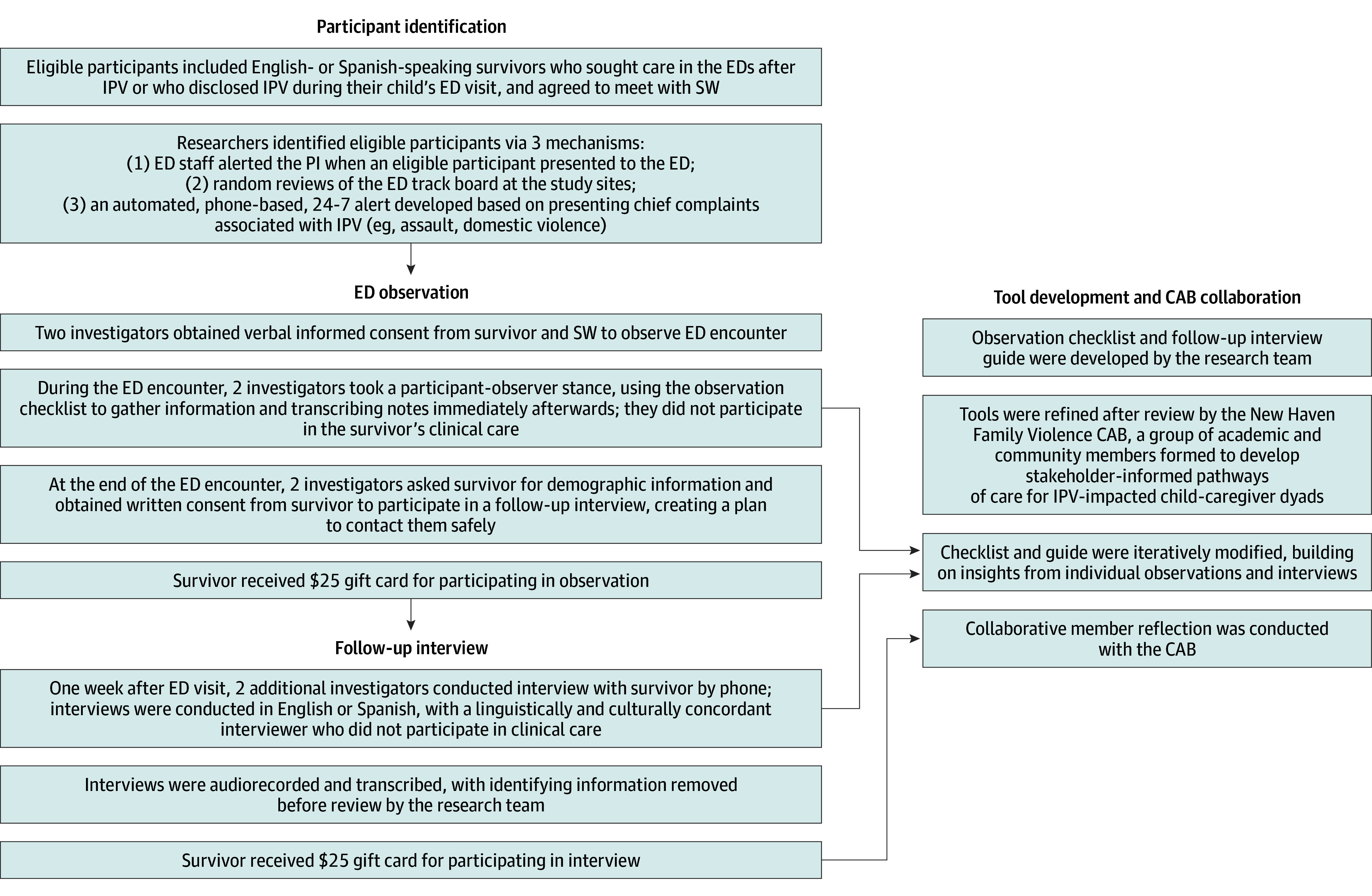
Eligibility, Recruitment and Data Collection Overview of tool development, participant identification, and data collection procedures, including emergency department (ED) observation and follow-up interviews. Tools were refined in collaboration with a Community Advisory Board (CAB). Participants received $25 gift cards for each study activity. IPV indicates intimate partner violence; SW, social worker.

### Underlying Epistemology

Our work is grounded in a constructivist paradigm, which purports that reality exists within and between social beings and that knowledge is constructed through experience and interaction.^14^ Because researchers participate in knowledge construction, we practiced reflexivity (eTable in Supplement 1) and acknowledged that our perspectives influenced data collection and analysis.

### Focused Ethnography

Focused ethnography involves targeted observations of specific interactions rather than prolonged observation in natural settings typical of traditional ethnography. It uses multiple data sources (interviews, observations) to capture complex processes and has been applied to study the nuanced interplay of social and cultural factors influencing health care delivery and patient-clinician interactions.

### Setting

We conducted a focused ethnographic study from November 16, 2022, to June 30, 2024, across 3 EDs in a single health system: 1 pediatric academic ED, 1 general academic ED, and 1 general community ED serving children and adults, with annual volumes ranging from 25 000 to 100 000 patients. We included IPV-related encounters for medical, psychological, or safety concerns, in which SWs were involved. In a prior study performed in the same health system, including the 2 general EDs, approximately 75% of 75 survivors presenting after IPV accepted ED SW consultations annually.^15^

### Participants and Data Collection

Participants included survivors who preferred to speak English or Spanish and who sought care after IPV. The research team developed a checklist for observations and interview guide that were refined after review with a Family Violence Community Advisory Board (CAB) of academic and community members.^16^ CAB members provided feedback on checklist context and flow and interview guide questions. Before starting the study, the interview guide underwent a final review by an ED SW and an IPV survivor to ensure the questions were trauma informed. Race and ethnicity were self-reported, with survivors identified as Asian, Black, Hispanic, White, multiracial, or other (including American Indian or Alaska Native and Native Hawaiian or Other Pacific Islander). These data were collected to ensure capture of a diverse range of IPV survivor experiences and to situate these experiences within broader social and structural contexts, including inequities in emergency care.

### Observations

Observations allowed the research team to identify clinical behaviors and contextual factors that survivors might not recall or articulate in interviews. Two investigators (G.T. and B.O.) purposively sampled across ED types and ensured adequate representation of Spanish-speaking survivors. After obtaining verbal informed consent from the survivor and the SW, they used the checklist (Table 1) to guide detailed note taking during observations of SW-survivor interactions occurring in English or with bilingual clinicians or interpreters, focusing on ED context and the content of the SW-patient interaction.^17^ Observers then obtained written consent from the survivor to participate in a follow-up phone call interview.

**Table 1.** Observation Checklist

Area of focus	Questions and/or prompts
Survivor situation and expressed needs	1. What happened to bring the patient to the ED? What led them to seek safety?
	2. What needs do the survivors or parents disclose?
Assessment and support offered during encounter	3. How is the safety of children assessed? How is reporting to CPS approached? What is the survivor’s reaction to this?
	4. How is support provided (emotional, informational, referrals)? Is there discussion about safety and community resources?
Barriers and facilitators to engagement	5. How does the social worker build engagement (eg, body language or communication style)? Are there any barriers to engagement present (eg, preconceived notions, projecting preferences, judgmental attitudes)?
	6. What ED-level barriers to engagement are present (eg, space, timing, privacy, general chaos)?
	7. What personal barriers to engagement do patients face (eg, fear of CPS, minimizing IPV, shame, safety concerns)?

Abbreviations: CPS, child protective services; ED, emergency department; IPV, intimate partner violence.

### Follow-Up Interviews

Follow-up interviews captured survivors’ interpretations of their experiences and aspects of the visit that were not outwardly observable. Two investigators (including D.C.) who were bilingual in English and Spanish performed follow-up interviews in English or Spanish, exploring survivors’ reflections on their ED visits. The interviews focused on the issues the survivors sought to address during their ED visits and beneficial or challenging aspects of the ED visit (Table 2). Interviews were audio-recorded and transcribed; those conducted in Spanish were first transcribed and then translated into English by the professional transcription service before review. Survivors received a $25 gift card for the observation and an additional $25 for the interview.

**Table 2.** Interview Guide

Area of focus	Questions and/or prompts
Survivor situation and priorities	1. What were the main things you were hoping to address or get out of the ED visit? Recursive probe: From what I learned, you were really hoping to address [X, Y, Z]. How is that going?
	2. Tell me what has happened since your ED visit.
	3. What do you hope for yourself or your child in the next year? Probe: What would help you get to that goal?
Connection to IPV resources after ED visit	4. Were you able to follow up with any community resources, specifically DV resources?
	5. To the best of your knowledge, was a report made to CPS or DCF during your visit to the ED? If yes, probe: Tell me about that experience. Were you able to get support or resources through DCF?
Feedback on ED experience	6. Did you feel the providers met your needs? What was helpful to you (eg, resources, peace of mind about your child’s health)? Probe: What would have helped more during your evaluation in the ED?
	7. What could we have done to make your visit better for you?
	8. How can physicians, nurses, and social workers better help and support those affected by domestic violence in the ED?
Association of ED experience on future willingness to seek help	9. How do you feel about asking for help in the future if you experience violence at home? Probe: Has this experience (having yourself or your child checked by a physician) changed your willingness to ask for help?
	10. What are your thoughts about following up with DV resources after this visit?

Abbreviations: CPS, child protective services; DCF, Department of Children and Families; DV, domestic violence; ED, emergency department; IPV, intimate partner violence.

### Statistical Analysis

Consistent with constructivism, we iteratively collected and analyzed data and refined the checklist and interview guide as the study progressed. We started analysis with deductive codes informed by core principles of TVIC (Table 3), then created inductive codes based on emergent concepts. During the 18-month study period, we met after each observation and interview to expand, refine, and merge codes. After completing 28 observations and 13 interviews, our code lists were stable (ie, no new key concepts were emerging).^18,19^ One investigator (D.C.) then applied the final code lists to the full datasets. While observations and interviews were analyzed using separate codebooks, they were integrated to build a cohesive set of themes about survivors’ experiences and ED contextual factors regarding IPV-related interactions. In the final analysis phase, we met for 2 months to cluster codes into descriptive themes linked to TVIC principles, which served as an organizing framework. Qualitative software (ATLAS.ti, version 5.0 [ATLAS.ti Scientific Software Development GmbH]) facilitated data organization and retrieval.

**Table 3.** TVIC Alignment

Principle	Findings	
	TVIC aligned	TVIC misaligned
Recognizing the impacts of trauma and violence, including structural violence and inequities	Showing empathy and providing validation when survivor discloses past personal or familial exposure to violence, previous CPS involvement, mental illness, substance use disorder, poverty, or immigration concerns.	Lack of consideration about how survivors’ intersecting marginalized identities or past experiences may impact their experience seeking help.
Prioritizing emotional, physical, and cultural safety	Providing a comfortable, private room with minimal background noise, a bilingual/bicultural social worker, or effectively using a video interpreter.	Chaotic ED environment, hallway beds, long wait times, no discussion of visitor restrictions, or lack of electronic health record safety considerations.
Connectedness, collaboration, and choice	Using positive body language, validating words, identifying survivor priorities, and emphasizing survivor autonomy.	Relying on closed-ended questions, prioritizing the clinician’s concerns over the survivor’s, particularly when making reports to CPS, or repeatedly asking the survivor to recount their story.
Fostering capacity development	Ensuring survivors are equipped to address their holistic needs after discharge by teaching skills for safety planning and connecting them with community resources.	Failure to provide warm handoffs when referring survivors to services.

Abbreviations: CPS, child protective services; ED, emergency department; TVIC, trauma- and violence-informed care.

We incorporated numerous checks of trustworthiness. Notably, methodological triangulation deepened our understanding and ensured that survivors’ voices contextualized the observed interactions. We maintained a codebook with definitions and an audit trail of our coding process. To capture diverse experiences, we conducted observations at all times, including nights and weekends. We collected data until we reached thematic sufficiency.^18,19^ Finally, we performed a collaborative reflexive exercise^20,21^ in which we checked our interpretation of the data with the CAB. Following an educational session about TVIC, CAB members reviewed findings and affirmed that, based on their lived experiences and those of their clients or patients, findings captured the complexities of survivor experiences.

## Results

We recruited 31 survivors (29 female [94%], 1 male [3%], and 1 other [3%]; mean [SD] age, 29.0 [8.7] years). Twenty-four survivors (77%) preferred English, 6 (19%) preferred Spanish, and 1 (3%) preferred another language. In terms of race and ethnicity, 7 (23%) were Black; 15 (48%), Hispanic; 4 (13%), White; 4 (13%), multiracial; and 1 (3%), other race or ethnicity (Table 4). Among these participating survivors, we conducted 29 observations of interactions between survivors and 18 different SWs. Of the 19 survivors who consented, 13 participated in follow-up interviews approximately 1 week after ED visits, after as many as 3 contact attempts. Twenty-one survivors presented to the general EDs following IPV-related injuries, while in the pediatric ED, 10 survivors disclosed IPV during a child’s medical or behavioral health visit. Most survivors described severe physical violence (eg, strangulation), often co-occurring with other types of violence (psychological, sexual, financial, technological, and/or legal).

**Table 4.** Participant Demographic Characteristics

Characteristic	Emergency department type, No. (%)			Total (N = 31), No. (%)^a^
	General academic (n = 16)	Pediatric (n = 10)	General community (n = 5)	
Age group, y				
<18	0	1 (10)	0	1 (3)
18-23	6 (38)	3 (30)	1 (20)	10 (32)
24-29	3 (19)	2 (20)	1 (20)	6 (19)
30-40	6 (38)	3 (30)^b^	2 (40)	11 (35)
>40	1 (6)	1 (10)	1 (20)	3 (10)
Age, mean (SD), y	29.5 (9.5)	27.0 (7.9)	31.2 (8.3)	29.0 (8.7)
Gender				
Female	16 (100)	10 (100)	3 (60)	29 (94)
Male	0	0	1 (20)	1 (3)
Other	0	0	1 (20)	1 (3)
Race and ethnicity				
Asian	0	0	0	0
Black	6 (38)	1 (10)	0	7 (23)
Hispanic	6 (38)	6 (60)	3 (60)	15 (48)
White	2 (13)	0	2 (40)	4 (13)
Multiracial	1 (6)	3 (30)	0	4 (13)
Other	1 (6)	0	0	1 (3)
No. of children				
0	7 (44)	2 (20)	1 (20)	10 (32)
1-2	4 (25)	5 (50)	3 (60)	12 (39)
3-4	4 (25)	2 (20)	1 (20)	7 (23)
>4	1 (6)	1 (10)	0	2 (6)
Language preference				
English	14 (88)	7 (70%)	3 (60)	24 (77)
Spanish	1 (6)	3 (30%)	2 (40)	6 (19)
Other	1 (6)	0	0	1 (3)
CPS reports when children living in home^c^				
Yes	3 (60%)	4 (50)	3 (75)	10 (59)
No	2 (40%)	4 (50)	1 (25)	7 (41)
Participation in interview				
Yes	3 (19)	8 (80)	2 (40)	13 (42)
No	13 (81)	2 (20)	3 (60)	18 (58)

Abbreviation: CPS, child protective services.

^a^ One participant underwent observations during 2 separate encounters.

^b^ Two participants consented to and participated in follow-up interviews; however, because the social worker consultations occurred before the observer was notified, they did not participate in observations.

^c^ Measured as number (percentage) of encounters.

Themes are organized around TVIC principles and summarized in Table 3. Principles include recognizing the impacts of trauma; prioritizing safety, connectiveness, and choice; and fostering capacity development. Observational descriptions of SW-survivor interactions and ED context and interview data are presented to illustrate how principles are reflected in practice.

### Recognizing the Impacts of Trauma and Violence

A TVIC approach recognized how past trauma and intersecting marginalized identities often shaped survivors’ experiences of seeking help for IPV. An observer of a Black participant in her 20s in the general academic ED noted:

[Survivor] grew up in foster care, was raped, and had domestic violence in her past relationships, resulting in significant anxiety and PTSD [posttraumatic stress disorder]. After 2 years of homelessness, she recently got an apartment, but worried she would get kicked out since [the abuser] kept coming back. She declined involving law enforcement because she said police might not believe her, they didn’t take her seriously in the past.

Immigrant survivors discussed concerns about deportation, housing, or separation from their children. One Hispanic survivor in her 20s in the pediatric ED who was a recipient of Deferred Action for Childhood Arrivals (DACA) shared that her partner threatened to take their children if she sought help in the ED. Another Hispanic survivor in her 20s in the general academic ED described not having a support system in the US and having to return to her in-laws’ home, where the violence took place.

SWs acted in alignment with TVIC by actively listening and responding with empathy. When the SW allayed the survivor’s fear that her child would be removed, assuring her that child protective services (CPS) would not be called due to her DACA status, the survivor described that conversation as “feeling like a therapy session,” increasing her willingness to seek help after the visit. The survivor living with her in-laws returned to her home but expressed gratitude that the SW had provided her with options for emergency housing and connected her to IPV services.

### Prioritizing Safety

#### Creating Safe Spaces

The physical environment influenced how survivors engaged with services and disclosed their experiences. Survivors appeared most comfortable and forthcoming when encounters took place in calm, relatively quiet environments. In one pediatric academic ED interaction involving a biracial survivor in their late 20s, the observer’s description was: “SW sat down in a private room. Mom comfortable on bed with baby.” This survivor endorsed a positive experience in a follow-up interview: “Everything was great, nothing could have gone better … the conversation was private and not rushed.”

However, this was not always the case, particularly in the general EDs. In one general academic ED encounter involving a Hispanic participant in her 20s, the observer’s description was: “Curtain room with numerous loud voices. Right outside her room is a behavioral health patient loudly yelling curses/screaming expletives.” Background noise and interruptions often resulted in survivors having to repeat their stories numerous times, and led one Black survivor in her 20s in the general academic ED to give up on the encounter entirely:

Interruptions with providers coming in and out, calling for imaging studies, tech coming to take her to imaging. Ultimately [she] decided to leave and not wait for shelter or the rest of her work-up. [She was observed to be] yelling at ED staff prior to leaving.

SWs often found creative ways to optimize chaotic environments to provide TVIC-aligned care:

[SW] moved the survivor from a recliner in the [academic ED] hallway to a quiet private room with a couch. The survivor showed relief and appreciation; she “wanted to know if she could stay in there a bit longer after the interview.”

Similarly, some SWs found ways to successfully separate children when discussing IPV. For example, in the pediatric ED, they partnered with child life specialists to engage older children elsewhere or arranged for a sitter to speak with the survivor privately.

#### Limiting Abuser Access

Safety was compromised when abusers remained in contact with the survivor. During one academic ED visit, a White survivor in her 50s received threatening messages from her abuser, such as, “I’m coming after you.” In some cases, visitor restrictions helped improve safety. After an academic ED visit, one Hispanic Spanish-preferring survivor in their 20s emphasized, “abusers should not be allowed in the ER, not good for family.” Ensuring medical record confidentiality also promoted trust and engagement. During an interview, one multiracial survivor in their late 20s seen in the pediatric academic ED mentioned, “[I] really liked that the notes would be hidden so the baby’s father wouldn’t be able to see what was discussed during the visit.”

####  Honoring Language Preferences

Language concordance promoted cultural safety by allowing survivors to express themselves, feel understood, and engage in care without communication barriers. For a Hispanic Spanish-preferring survivor in her 20s in the pediatric academic ED, an observer noted, “SW speaks Spanish, and the connection is incredible … culturally responsive. She’s so in touch with the mom, and the mom automatically turns to her.” When interpreters were required, thoughtful use of translation services enhanced trust. With a Hispanic Spanish-preferring participant in her 40s in the general community ED, the observer noted, “SW made sure the translator machine camera was not facing the victim, to respect her privacy and religion.”

In contrast, communication barriers led a Spanish-preferring survivor in his 20s in the general community ED to perceive refusal of care:

After the initial meeting with SW, the victim went into the waiting room, believing they were refusing him care. [Hospital staff] brought him back [to the ED care area] from the hallway, where he was talking to one of the policemen who spoke Spanish.

Environmental challenges further complicated communication for non-English speakers. One observer noted the “[general academic] ED was busy and loud, making it difficult to hear the translator.”

### Connectedness, Collaboration, and Choice

A TVIC-approach emphasized building trusting relationships, sharing power, and supporting survivors’ priorities. Survivors were more engaged when the SW communicated empathetically, validated priorities, and respected autonomy. For a Hispanic survivor in her late 20s in the general academic ED,

SW made eye contact throughout. He laughed when the patient joked. He used validating language, saying, “you are a strong and intelligent woman,” and emphasized it was her decision to call the police or the IPV hotline.

Parenting was a priority in survivors’ decision-making, as illustrated in an interview of a Hispanic, Spanish-preferring survivor in their 20s seen in the pediatric academic ED, who noted: “My daughter is the reason I left the violence. My mom told me that I have to leave the violence and first comes our children…. My daughter will keep me strong.” SWs encouraged survivors’ efforts to protect their children and leverage their support systems.

In contrast, observers noted that survivors often disengaged when communication focused on the clinician’s priorities. For a Hispanic survivor in their 30s in the general academic ED:

SW used phrases such as: “this is what I need you to do,” “we’re recommending you…,” “try to focus,” “try to remember.” SW asked lots of questions and sometimes interrupted before the patient answered, instead of letting the patient tell the story in one sequence…

Conversations about CPS reporting were sometimes directive, as demonstrated in one TVIC-misaligned interaction with a Black survivor in her 20s in the general academic ED:

SW learned that the patient’s younger sister was present during the incident. Immediately, he asked questions about her age, name, and whereabouts. Patient described her sister had hidden in the bathroom during the incident, remaining safe. SW immediately stated, “We are mandated reporters, I have to call DCF [Department of Children and Families].” Patient teared up, seemed worried, and seemed to shut down.

Some SWs, in contrast, used empathetic language to remain connected during conversations about CPS, as in a case with a multiracial survivor in her late 20s in the pediatric academic ED:

SW gently described that she would have to call DCS since her daughter had witnessed so much of the violence. She told the mom, “You did all the right things, I’m grateful you called the police,” but I do have to call DCS. Mom remained engaged.

### Fostering Capacity Development

SWs helped survivors build practical skills and connected them to resources that addressed both immediate safety concerns and systemic needs. For a Hispanic survivor in her 30s in the pediatric academic ED, an observer noted that “SW demonstrated how to make her phone safe and discussed being careful what she disclosed to kids’ school so [the abuser] couldn’t track them down.” SWs assessed acute needs unrelated to IPV and provided resources for accessing public benefits, legal aid, transportation, emergency shelters, mental health, and pregnancy-related care.

SWs offered warm handoffs, in which they personally connected survivors to another service with support or introduction. In one scenario, the observer noted, “SW called IPV services and did the intake with the survivor during the visit, using an interpreter.” However, this was not the case in every encounter. In follow-up interviews, a key strategy to improve care included offering warm handoffs or making the first contact for services such as mental health, police, and IPV advocacy.

## Discussion

This qualitative study is the first, to our knowledge, to examine the delivery of TVIC by SWs for IPV survivors in EDs. Using observational data and survivor interviews, we identified areas where care aligned with TVIC and highlighted opportunities for improved alignment.

Survivor-centered care, a key aspect of TVIC, involves respecting survivors’ autonomy and supporting their self-identified priorities.^11^ We found that empathetic communication and affirming survivors’ resilience enhanced engagement, findings consistent with evidence linking survivor-centered practices and safety-related empowerment.^22,23,24,25^ Conversely, a prescriptive approach could undermine trust and discourage future help-seeking.^8^

Capacity building ensured survivors could meet their needs after discharge. While SWs provided resources for employment, immigration, parenting, and other needs, information alone was insufficient. Previous studies highlight inadequate coordination between hospitals and community agencies.^26,27,28^ Embedding dedicated IPV advocates within hospitals who can help survivors navigate services and may strengthen follow-up care, improve access to resources, and reduce IPV.^26,27,29,30^

At the core of TVIC is an understanding that survivors’ experiences of trauma are shaped by broader systemic inequities. Our results support literature demonstrating that immigrant survivors face language, cultural, and legal barriers, while Black survivors may feel unsafe with law enforcement.^31,32,33^ Poverty further compounds IPV, leading to financial dependence due to scarce resources such as housing.^34,35^ Our findings underscore the need for clinicians to adopt an intersectional approach to IPV care, recognizing and addressing personal and structural barriers that intersect to influence survivor experiences seeking and accepting care.

The ED environment and workflow impacted survivor experiences. Our findings support previous studies highlighting how crowded, noisy environments exacerbate stress for patients after violent injuries.^36,37^ Although the pediatric ED often offered quiet, private rooms and child life support, those in the general EDs frequently received care in hallway beds with significant background noise. Health systems could mitigate these challenges by establishing private consultation spaces and assigning personnel to support children during IPV discussions.^8^ Policies should recommend shared decision-making on clinician- or SW-initiated visitor restriction, coordinated with leadership and security to ensure safety. Accreditation standards should incentivize these practices so that privacy, access, and noise reduction are treated as safety issues, not amenities.

Cultural safety, another aspect of TVIC, is critical for engagement.^38^ Our findings aligned with literature demonstrating that survivors expressed difficulty trusting clinicians who are not racially or culturally concordant.^39^ Language justice sees language access as a right, emphasizing accurate interpretation and removing power imbalances that can coerce survivors.^40,41^ EDs should fund strong language services, provide qualified interpreters, and train staff in culturally responsive communication to prevent coercion and build trust.^42,43^

Policies emphasizing digital safety are particularly relevant due to the 21st Century Cures Act. While open notes may enhance patient engagement, they endanger IPV survivors if abusers gain unauthorized access to their records.^44,45^ Policies should recommend shared decision-making to ensure safe documentation and detail when to invoke exceptions to information sharing, the use of protected note types, or the omission of IPV-related diagnosis codes, viewable by the abuser.^46,47^

Our findings also highlight the need for clearer guidance on reporting to CPS. At times, SWs in our study made incorrect statements about reporting mandates, did not provide clear communication about limits of confidentiality, or failed to communicate empathetically, potentially contributing to patient harm. While childhood IPV exposure poses serious health impacts, fear of reporting to CPS may serve as a barrier to seeking help and escalate IPV or lead to legal sanctions.^48,49,50,51^ Clinicians should consult state laws and local policies^52^ and assess how a child was harmed by the IPV. When reporting is necessary, policies should encourage clinicians to provide clear communication about confidentiality, include survivors in the reporting process, name the abuser, and refer to community resources.^50,53^ If the survivor is undocumented, referral to IPV advocacy may be especially critical for safety.^47^

Future work should examine long-term outcomes of TVIC within ED settings, including the effects on safety planning, health outcomes, and engagement with services. Systematic investigations of how ED layout and environment impact survivor perceptions of safety are critical and may justify institutional infrastructure investments. Finally, while emerging evidence suggests hospital-based IPV advocates may improve care, further research should examine their effectiveness in reducing IPV and improving access to resources.

### Limitations

This study has some limitations. First, researcher presence may have influenced behavior (observer effect); however, this aligns with a constructivist paradigm, where knowledge is socially constructed through interactions between researchers and participants. Second, although we observed many interactions with individual SWs, experiences within a single ED visit vary, with both supportive and harmful interactions from multiple staff members. We did not assess the cumulative effect of those mixed experiences or whether a single positive encounter mitigated the impact of negative ones. Third, our findings reflect the experiences of a small sample of participants with SW contact and may not be directly applicable beyond the study sites, although the inclusion of community, academic, and pediatric EDs serving diverse populations supports broader relevance. To enhance transferability,^54^ we also documented rich observational descriptions and used TVIC for conceptual grounding. Finally, while we included Spanish-preferring survivors, we did not capture perspectives from other minority racial and linguistic groups.

## Conclusions

In this qualitative study, alignment with TVIC occurred when clinicians offered empathetic, survivor-centered care, acknowledged systemic inequities, and ensured safety. However, TVIC was hindered by limited privacy, language barriers, and directive communication. Without TVIC, EDs risk retraumatizing survivors and missing key opportunities for meaningful intervention.

## Notes

Supplement 1. **eTable.** Reflexivity Statement

Supplement 2.
Data Sharing Statement
