# Supplementary material for: Trauma- and Violence-Informed Care Practices in the Emergency Department for Survivors of Intimate Partner Violence
Source: JAMA Netw Open. 2026 Mar 3;9(3):e260034. doi: 10.1001/jamanetworkopen.2026.0034 (PMC12958084; doi:10.1001/jamanetworkopen.2026.0034)
Supplement: Supplement 1. — eTable. Reflexivity Statement [file jamanetwopen-e260034-s001.pdf]

Supplementary Online Content

Tiyyaura G, Abeyaratne D, Asnes A, et al. Trauma- and violence-informed care practices in the emergency department for survivors of intimate partner violence. *JAMA Netw Open.* 2026;9(3):e260034. doi:10.1001/jamanetworkopen.2026.0034

eTable. Reflexivity Statement

This supplementary material has been provided by the authors to give readers additional information about their work.

© 2026 Tiyyaura G et al. *JAMA Network Open*.

eTable. Reflexivity Statement

Our research team brought a diverse range of professional, personal, and social identities to this study, which shaped our approach to data collection, analysis, and interpretation.<br><br>We recognize that our identities—particularly in terms of race, professional role, and lived experience—may have influenced how we understood and interpreted survivors' experiences, especially those involving systemic harm, structural violence, and trust in institutions. We engaged in ongoing reflection to identify and mitigate potential biases, centering survivors' voices throughout the analysis. By integrating clinical, advocacy, and survivor-informed perspectives, we sought to ensure that the findings reflected a trauma- and violence-informed lens grounded in both empathy and accountability to those most affected.	
**Study Team Member**	**Personal Reflexivity Statement**
AF	Caucasian female advocate and leader in Connecticut's Coalition Against Domestic Violence, brought a critical advocacy- and policy-informed perspective to the coding team.
BO	Caucasian female research assistant with prior qualitative research experience with IPV survivors who served as an observer and coder.
DA	Asian female medical student contributed to data analysis and interpretation, bringing a lens informed by academic inquiry and prior qualitative research experience with IPV survivors.
DC	Multi-racial female (African American, Indigenous, and Caucasian) research assistant and master's of social work student, conducted interviews and brought frontline insight to survivor narratives.
GT	Asian female pediatric emergency medicine physician with expertise in IPV, contributed both clinical and research perspectives and served as an observer where she was able to use her perspective as an emergency medicine physician to better understand context and clinical care. She also coded the data.
ME	Hispanic female bilingual, bicultural research assistant trained in qualitative research with vulnerable populations who conducted the interviews of Spanish-preferring survivors.
MG	Caucasian female nurse researcher and leader of the hospital's violence intervention program, provided a systems-level healthcare lens on the coding team.
PS	Caucasian female program manager of the Yale Child Abuse Program, contributed knowledge of child welfare systems and their intersection with IPV on the coding team.

© 2026 Tiyyaura G et al. *JAMA Network Open*.
