# Supplementary material for: Trauma- and Violence-Informed Care Practices in the Emergency Department for Survivors of Intimate Partner Violence
Source: JAMA Netw Open. 2026 Mar 3;9(3):e260034. doi: 10.1001/jamanetworkopen.2026.0034 (PMC12958084; doi:10.1001/jamanetworkopen.2026.0034)
Supplement: Supplement 2. — Data Sharing Statement [file jamanetwopen-e260034-s002.pdf]

Data Sharing Statement

Tiyyagura. Trauma- and Violence-Informed Care Practices in the Emergency Department for Survivors of Intimate Partner Violence. *JAMA Netw Open*. Published March 03, 2026. doi:10.1001/jamanetworkopen.2026.0034

Data
Data available: No

Additional Information
Explanation for why data not available: There is risk to survivors of violence and their children in making their data available
